# Ideal birth spacing between the first and second child among reproductive-aged women in Shandong Province, China

**DOI:** 10.3389/fpubh.2026.1842402

**Published:** 2026-07-13

**Authors:** Mengjun Cao, Baotao Cheng, Zihan Zhou, Hongyan Hao, Ying Zhao, Hongmei Du, Qiang Wang

**Affiliations:** 1 College of Public Health, Shandong Second Medical University, Weifang, Shandong, China; 2 Department of Quality and Safety Management, Public Health Clinical Center Affiliated to Shandong University, Jinan, Shandong, China; 3 Department of Histology and Embryology, College of Basic Medical Sciences, Shandong Second Medical University, Weifang, Shandong, China; 4 Department of Epidemiology, Shandong Second Medical University, Weifang, Shandong, China

**Keywords:** fertility policy, ideal birth spacing, influencing factors, reproductive-aged women, social ecological model

## Abstract

**Objectives:**

This study aims to assess the ideal birth spacing between the first and second child among reproductive-aged women in Shandong Province and to examine its influencing factors using the Social Ecological Model. It was hypothesized that women’s ideal birth spacing is influenced by factors at the individual, family, and sociocultural levels, with these factors exerting different effects across categories of ideal birth spacing.

**Methods:**

A multistage random sampling method was employed to obtain a sample of 2,090 women. Descriptive statistics, univariate analysis and a generalized ordered logit model (gologit) were employed to identify factors associated with ideal birth spacing. Average marginal effects (AMEs) were calculated to interpret the magnitude of significant associations.

**Results:**

Among 2,090 participants, 46.32% preferred medium ideal birth spacing (3–4 years), 21.10% preferred short (0–2 years) and 32.58% preferred long spacing (≥5 years or no specific preference). Univariate analysis revealed that age, education level, marital status and fertility-related factors were significantly associated with ideal birth spacing (*p* < 0.05). Multivariable analysis revealed that the perceived impact of childcare challenges, impact of social and family expectations, as well as marital status, occupation, self-rated health, intended number of children, only-child status, household size, residence, monthly household income and awareness of fertility policy, were significantly associated with ideal birth spacing (*p* < 0.05).

**Conclusion:**

Women’s ideal birth spacing is shaped by multiple factors across individual, family, and sociocultural levels, with their effects differing across short, medium, and long spacing preferences. Policies aimed at reducing childcare burdens, improving policy awareness, and providing stage-specific support may help women better achieve their preferred reproductive timing.

## 1 Introduction

Fertility rates have been declining globally. The Population Division of the United Nations Department of Economic and Social Affairs (UN DESA) reported that more than half of countries and regions had a total fertility rate (TFR) below 2.1 children per woman (1, 2). China exhibits particularly low fertility compared to this global trend. Data from the National Bureau of Statistics of China reported that births have continued to decline, with the natural population growth rate reaching −0.99‰ in 2025 (3). In response to declining fertility, China implemented the universal two-child policy (UTCP) in 2016. Although the policy led to a temporary rise in births, the long-term decline in fertility continued (4). However, the problem was reflected not only in the decline in the number of children born but also in notable changes in the timing of childbearing. Longer birth spacing represented a key aspect of these timing-related changes (5, 6). Consistent with this, previous studies suggested that longer birth spacing has partly contributed to the recent decline in fertility in China (6). In this context, analyzing birth spacing and its influencing factors has significant academic value and may inform future research on fertility decisions among reproductive-aged women.

Existing studies on birth spacing have largely focused on the actual fertility outcomes (7, 8). However, prior to childbirth, reproductive-aged women typically form subjective expectations about birth spacing, defined as the preferred spacing between the first and second child, hereafter referred to as ideal birth spacing in this study (9). In research on fertility behavior, such subjective preferences are generally classified as fertility desires. These desires are important cognitive factors distinct from actual fertility outcomes and play a key role in fertility decisions (9, 10). Further studies indicated that fertility desires encompass not only the desired number of children but also expectations for birth timing and spacing (11)^.^ As a key dimension of such desires, ideal birth spacing reflects individuals’ comprehensive assessment of health, family resources and social support conditions (12). Thus, focusing on ideal birth spacing provides a critical analytical window for understanding how fertility intentions are translated into behavior. But these expectations cannot be fully captured by objective indicators (such as whether a birth occurs or the specific timing of births) and research specifically addressing the factors of ideal birth spacing remains limited. Therefore, it is necessary to study ideal birth spacing separately. Overall, analyzing the ideal birth spacing between the first and second child among reproductive-aged women and its influencing factors can help reveal the cognitive mechanisms underlying fertility decisions and provide scientific evidence to guide the optimization of fertility support policies, thereby helping to address the challenge of low fertility in China.

This study selected Shandong Province as the research setting. As one of China’s most populous provinces, it is highly representative in terms of population size, structural demographic changes and fertility challenges, which may provide valuable empirical evidence for informing regional and national fertility policy. In addition, considering the multilevel influences on perceptions of ideal birth spacing, including individual, family, social and policy environment, this study adopted the Social Ecological Model (SEM) as the theoretical framework (13, 14). The SEM emphasizes that individual decisions are shaped by interactions across five levels: individual, interpersonal, organizational, community and policy factors. This framework allows for a systematic examination of how perceptions of ideal birth spacing are formed. Accordingly, this study aims to examine ideal birth spacing between the first and second child among reproductive-aged women in Shandong Province and to analyze the key social, economic, health and cultural factors influencing these expectations. The findings may provide scientific evidence to support fertility policy optimization, promote more balanced population development and inform similar studies in other regions.

## 2 Methods

### 2.1 Participants

This was a population-based cross-sectional design study. This study investigated reproductive-aged women in Shandong Province in 2024. The participants of this study were reproductive-aged women aged 18 to 45 years who were registered residents of Shandong Province. According to international standards, reproductive-aged women include all females aged 15–49, independent of marital status (15). Based on the Chinese legal definition of adulthood, this study set 18 years as the minimum age to ensure that all participants were adults (16). Women over 45 face higher childbirth risks, lower fertility and declining ovarian function. According to previous findings, women aged 40–49 have limited contribution to Chinese fertility (17). Therefore, this study set 45 years as the maximum age.

The inclusion criteria were: (1) reproductive-aged women with household registration in Shandong Province; (2) women aged 18–45 years; (3) women who agreed to participate in the study and were able to complete the questionnaire. The exclusion criteria were: (1) reproductive-aged women without household registration in Shandong Province; (2) women outside the age range of 18–45 years; (3) women who were not willing or able to participate in the survey.

### 2.2 Sample size

The required sample size (*N*) was determined using the standard formula for estimating a single proportion (18).


$$N=\frac{Z_{\alpha /2}^{2}\cdot P(1-P)}{\delta^{2}}$$

In this formula, *N* represents the required sample size, Z_α/2_ is the standard normal critical value for a two-sided significance level α, *P* is the estimated proportion and δ is the allowable margin of error. A 95% confidence level was adopted (Z_α/2_ = 1.96). In the absence of prior population-based estimates, *P* was conservatively set at 0.5 to maximize the required sample size (19). The allowable margin of error (*δ*) was set at 0.05 (20). Based on these parameters, the minimum required sample size was calculated to be 384.

To account for the design effect of multistage sampling (Design Effect, DEFF = 2) and an anticipated 20% rate of invalid questionnaires (21, 22), the sample size was further adjusted. First, the sample size was multiplied by the design effect:


$$N_{\text{DEFF}}=N\times \text{DEFF}$$

Next, the sample size was adjusted for the expected proportion of invalid or non-response questionnaires (*r* = 0.20):


$$N_{\text{final}}=\frac{N_{\text{DEFF}}}{1-r}$$

Substituting the values from this study (*N* = 384, DEFF = 2, *r* = 0.20):


$$N_{\text{DEFF}}=384\times 2=768$$

$$N_{\text{final}}=\frac{768}{1-0.2}=960$$

Thus, the final required sample size was 960.

### 2.3 Sampling strategy

A multi-stage random sampling approach was used in this study. The 16 cities in Shandong Province have different demographic characteristics attributed to divergent social, economic and cultural conditions. Thus, in the first phase, the 16 cities were stratified into three clusters by similar economic development levels (e.g., per capita GDP), educational attainment, cultural backgrounds, and geographic proximity. In the second phase, three cities were chosen randomly from each of the predefined clusters as representative units, yielding nine sample cities in total. Random sampling ensures that each city within the same group has an equal probability of being selected, thereby ensuring the representativeness of the sample. In the third phase, one county or district was randomly selected from each sample city, resulting in nine county or district units. In the fourth phase, one subdistrict and one township were randomly selected from each sample county or district, yielding nine subdistricts and nine townships. In the fifth phase, one urban community and one rural village were randomly selected from each sample subdistrict and township, providing 18 final survey sites (9 urban and 9 rural). Reproductive-aged women who met the inclusion criteria and lived in the sampled communities or villages were included. At each stage, the number of urban communities and rural villages was proportionally allocated according to the actual number of reproductive-aged women in each area, ensuring balanced urban–rural representation and overall sample representativeness. A total of 2,422 reproductive-aged women participated, and 2,090 valid questionnaires were retained, representing an effective response rate of 86.3%.

### 2.4 Variables included

Data for this study were collected through a fertility-related questionnaire survey conducted among reproductive-aged women in Shandong Province. The questionnaire was originally developed by our research team for a broader study on second-child fertility intentions and related perceptions under the current fertility policy context. The present study focuses on the perceptions of ideal birth spacing among reproductive-aged women. The questionnaire items were developed based on a comprehensive literature review and theoretical framework. Content validity was assessed by experts, and the instrument was further refined through a pilot survey. The questionnaire demonstrated good psychometric properties (KMO = 0.940, Bartlett’s test *p* < 0.001, Cronbach’s *α* = 0.955), supporting its appropriateness for the current analysis.

The dependent variable was the ideal birth spacing among reproductive-aged women. It was assessed using the question: “In your opinion, what length of time between two births is appropriate?,” with three response options: “0–2 years,” “3–4 years” and “≥5 years or no specific preference.” The “≥5 years” and “no specific preference” options were grouped into the same category, as both reflect the theoretical ordering of the outcome and a relatively lower preference for short spacing in childbearing. In this study, for clarity in result presentation and interpretation, these categories were further labeled as short, medium and long ideal birth spacing. Higher categories indicated longer perceived ideal birth spacing. For women without prior childbirth experience, this question reflected their anticipated or hypothetical perceptions of ideal birth spacing. This variable was treated as an ordinal outcome in the analyses.

The independent variables were selected based on the SEM. The SEM is a multilevel framework, and its hierarchical structure can be appropriately adapted to the specific research context (23). It initially comprised five levels: individual, interpersonal, organizational, community, and policy (13). In this study, the five levels were restructured into three analytical levels based on theoretical consistency and analytical focus. The interpersonal level and the family-related components of the organizational level (e.g., household size) were combined into the “interpersonal/family level.” Because the family serves as the smallest unit of social interaction and possesses organizational characteristics, these two domains exhibit considerable overlap in shaping fertility preferences. The community and policy levels were merged into the “social/environmental level,” as community context and broader policies together constitute the external social environment shaping individual fertility decisions. This three-level structure preserves the core logic of the SEM in conceptualizing fertility preferences as the result of multilevel interacting factors. The specific variables included at each level are detailed as follows.

At the individual level, variables included age, educational level, marital status, only-child status, occupation, self-rated health, intended number of children, perceived impact of fertility attitudes and career-fertility conflict (reflecting the tendency to prioritize either personal development or childbearing).

At the interpersonal/family level, variables included impact of childcare challenges, impact of social and family expectations, household size and monthly household income.

At the social/environmental level, variables included residence and awareness of fertility policy.

To reduce dimensionality and improve measurement stability, principal component analysis (PCA) was applied to three groups of related subjective perception variables to construct continuous composite indicators. These indicators capture the impact of fertility attitudes (traditional values, emerging parenting concepts, and emotional needs), the impact of childcare challenges (burdens across preschool, kindergarten, and primary school stages), and the impact of social and family expectations (normative pressures from family members and social networks). The first principal component was retained for each conceptual construct, including fertility attitudes (eigenvalue = 3.73, explained variance = 74.54%, loadings ranging from 0.39 to 0.47), childcare challenges (eigenvalue = 2.91, explained variance = 72.75%, loadings ranging from 0.44 to 0.53), and social and family expectations (eigenvalue = 3.99, explained variance = 79.81%, loadings ranging from 0.42 to 0.47). These variables captured contextual perceptions rather than outcomes and were included as independent predictors in the regression models. Detailed information on all items and their corresponding dimensions is provided in Supplementary Table 1.

### 2.5 Statistical method

Data were imported into Stata 18.0 software to establish a database. Descriptive analyses were initially conducted on the eligible data to summarize sociodemographic characteristics and other basic information. Continuous variables with a normal distribution were presented as means ± standard deviation (SD), while non-normally distributed continuous variables were presented as medians and interquartile ranges (IQR). Categorical variables were presented as frequencies and percentages (n, %). Univariate analyses were performed for each independent variable to preliminarily examine potential factors. For continuous variables, one-way ANOVA was used for normally distributed data, while the Kruskal-Wallis H test was employed for non-normally distributed data. Categorical variables were compared using the Chi-square test.

A generalized ordered logit model (gologit) was employed to examine factors associated with ideal birth spacing among reproductive-aged women. The model was estimated using the gologit2 command in Stata (24, 25). It was selected because the dependent variable is ordinal and the parallel-lines (proportional odds) assumption of the standard ordered logit model was violated. The gologit framework allows the proportional odds assumption to be relaxed for variables that violate it, providing a more flexible modeling strategy (26, 27). A multinomial logit model was not considered, as it ignores the ordinal nature of the outcome and yields less interpretable results. Average marginal effects (AMEs) were subsequently calculated for each independent variable to assess their effects on the probability of each ideal birth spacing category.

All statistical tests were two-sided. Statistical significance in univariate analyses was defined as *p* < 0.05. For the gologit model, all candidate variables were included based on theoretical considerations. A threshold of *p* < 0.10 was used to identify potential associations for exploratory interpretation, rather than strict hypothesis testing (28). This approach helped avoid prematurely excluding potentially relevant variables.

### 2.6 Quality control

To ensure data quality, several quality control measures were implemented. Firstly, the questionnaire was reviewed by experts and pre-tested to ensure clarity and logical consistency. Secondly, the sample size was appropriately expanded to enhance statistical power and account for potential non-response. Thirdly, a multistage random sampling design was strictly followed to ensure sample representativeness. Fourthly, investigators received standardized training and data entry was double-checked to minimize errors.

### 2.7 Ethics approval

The study was conducted in accordance with the Declaration of Helsinki (1964, most recently revised in 2013) established by the World Medical Association. This study was approved by the Ethics Review Committee of the Shandong Second Medical University (2023YX-139). All participants provided informed consent voluntarily.

## 3 Results

### 3.1 Univariate analysis of factors influencing ideal birth spacing between the first and second child among reproductive-aged women

A total of 2,090 valid questionnaires were collected and all analyses were based on these responses. Among participants, 21.10% preferred “0–2 years” (short ideal birth spacing), 46.32% preferred “3–4 years” (medium ideal birth spacing) and 32.58% preferred “≥5 years or no specific preference” (long ideal birth spacing). Univariate analysis showed that the following variables were significantly associated with ideal birth spacing: age (*H* = 23.36, *p <* 0.001), impact of fertility attitudes (*H* = 9.59, *p =* 0.008), education level (χ^2^ = 17.71, *p =* 0.007), marital status (χ^2^ = 40.53, *p <* 0.001), awareness of fertility policy (χ^2^ = 29.05, *p <* 0.001), occupation (χ^2^ = 42.59, *p <* 0.001), self-rated health (χ^2^ = 15.63, *p <* 0.001), intended number of children (χ^2^ = 25.70, *p <* 0.001), only-child status (χ^2^ = 19.53, *p <* 0.001), household size (χ^2^ = 31.81, *p <* 0.001) and residence (χ^2^ = 21.50, *p <* 0.001). The main results are presented in Table 1, while the full results are reported in Supplementary Table 2.

**Table 1:** Univariate analysis of factors influencing ideal birth spacing.

Characteristic	Category	Frequency (%)	Ideal birth spacing [*n* (%)/Median (IQR)]			χ^2^/H	*p*-value
			0–2 years	3–4 years	≥5 years or no specific preference		
Participates	–	2090 (100.00)	441 (21.10)	968 (46.32)	681 (32.58)	–	–
Age	–	2090 (100.00)	34 (27, 40)	35 (30, 40)	34 (28, 39)	23.36	<0.001^*^
Impact of fertility attitudes	–	2090 (100.00)	0.09 (−3.04, 2.09)	0.65 (−2.27, 2.27)	−0.65 (−3.04, 2.01)	9.59	0.008^*^
Educational level	Junior high school or below	191 (9.14)	33 (17.28)	77 (40.31)	81 (42.41)	17.71	0.007^#^
	High school/Vocational High school/Technical secondary school	274 (13.11)	60 (21.90)	118 (43.07)	96 (35.04)		
	College/Bachelor’s degree	1,416 (67.75)	305 (21.54)	658 (46.47)	453 (31.99)		
	Postgraduate and above	209 (10.00)	43 (20.57)	115 (55.02)	51 (24.40)		
Marital status	Unmarried	370 (17.70)	94 (25.41)	128 (34.59)	148 (40.00)	40.53	<0.001^#^
	Married with no children	185 (8.85)	58 (31.35)	80 (43.24)	47 (25.41)		
	Married with children	1,535 (73.44)	289 (18.83)	760 (49.51)	486 (31.66)		
Awareness of fertility policy	Incorrect	1,066 (51.01)	224 (21.01)	523 (49.06)	319 (29.92)	29.05	<0.001^#^
	Clear	886 (42.39)	183 (20.65)	409 (46.16)	294 (33.18)		
	Unclear	138 (6.60)	34 (24.64)	36 (26.09)	68 (49.28)		
Occupation	Civil servant, public institution or state-owned enterprise personnel	1,054 (50.43)	217 (20.59)	541 (51.33)	296 (28.08)	42.59	<0.001^#^
	Foreign/private enterprise personnel/Self-employed	221 (10.57)	63 (28.51)	83 (37.56)	75 (33.94)		
	Migrant worker/Peasant	228 (10.91)	46 (20.18)	103 (45.18)	79 (34.65)		
	Student	191 (9.14)	47 (24.61)	67 (35.08)	77 (40.31)		
	Freelance work	111 (5.31)	21 (18.92)	53 (47.75)	37 (33.33)		
	Other practitioners/Unemployed	285 (13.64)	47 (16.49)	121 (42.46)	117 (41.05)		
Self-rated health	Very healthy	867 (41.48)	216 (24.91)	366 (42.21)	285 (32.87)	15.63	<0.001^#^
	Suboptimal health	1,223 (58.52)	225 (18.40)	602 (49.22)	396 (32.38)		
Intended number of children	0	138 (6.60)	33 (23.91)	50 (36.23)	55 (39.86)	25.70	<0.001^#^
	1	977 (46.79)	207 (21.19)	424 (43.40)	346 (35.41)		
	2	901 (43.11)	177 (19.64)	464 (51.50)	260 (28.86)		
	≥3	74 (3.54)	24 (32.43)	30 (40.54)	20 (27.03)		
Only-child status	Yes	485 (23.20)	137 (28.25)	201 (41.44)	147 (30.31)	19.53	<0.001^#^
	No	1,605 (76.80)	304 (18.94)	767 (47.79)	534 (33.27)		
Household size	1–2	140 (6.70)	41 (29.29)	53 (37.86)	46 (32.86)	31.81	<0.001^#^
	3	686 (32.82)	180 (26.24)	306 (44.61)	200 (29.15)		
	4	672 (32.13)	124 (18.45)	308 (45.83)	240 (35.71)		
	5	331 (15.83)	55 (16.62)	168 (50.76)	108 (32.63)		
	≥6	261 (12.49)	41 (15.71)	133 (50.96)	87 (33.33)		
Residence	Urban areas	1,563 (74.83)	350 (22.39)	746 (47.73)	467 (29.88)	21.50	<0.001^#^
	Rural areas	527 (25.17)	91 (17.27)	222 (42.13)	214 (40.61)		

1. Kruskal-Wallis H (*); Chi-square test (^#^). 2. The full table containing all variables examined (including non-significant ones) is available in the Supplementary materials.

### 3.2 Multivariable analysis of factors influencing ideal birth spacing between the first and second child among reproductive-aged women

#### 3.2.1 Collinearity test of independent variables

The Variance Inflation Factor (VIF) values of the independent variables ranged from 1.04 to 2.12 (Supplementary Table 3). All values were well below the commonly used threshold of 10, indicating no evidence of serious multicollinearity among the variables. This supports subsequent gologit analysis.

#### 3.2.2 Model goodness of fit test

To compare the two models and evaluate their validity, Pseudo *R*^2^, the Akaike Information Criterion (AIC) and the Bayesian Information Criterion (BIC) were used to assess model goodness of fit. In regression modeling, larger Pseudo *R*^2^ values and smaller AIC and BIC values indicate better fit. As shown in Supplementary Table 4, the gologit model yielded a higher Pseudo *R*^2^ and lower AIC and BIC than the ordered logit model, demonstrating superior fit. These results indicate that the gologit model is more appropriate for examining factors influencing ideal birth spacing among women of reproductive age in Shandong Province. Accordingly, subsequent analyses focus on the regression results of the gologit model.

#### 3.2.3 Factors associated with ideal birth spacing estimated using generalized ordered logit models and average marginal effects

The model was estimated using a gologit framework with a backward cumulative (ascending) approach. This method yields two distinct cumulative contrasts: (1) short (0–2 years) versus medium/long (3–4 years and ≥5 years or no specific preference) spacing and (2) short/medium (combined 0–4 years) versus long (≥5 years or no specific preference) spacing. Two complementary metrics are reported in this section: cumulative odds ratios (ORs) from the gologit model, and category-specific average marginal effects (AMEs).

For ORs, values below 1 indicate higher cumulative odds of shorter spacing, whereas values above 1 indicate higher cumulative odds of longer spacing. AMEs quantify the change in predicted probability for a specific spacing category, reported as percentage points (pp) in the text (with raw decimal probabilities presented in tables). Positive AMEs indicate an increased probability of selecting the target category, and negative values indicate a decreased probability. All OR and AME estimates are presented in Tables 2, 3.

**Table 2:** Generalized ordered logit model estimates for ideal birth spacing.

Variables	Category	Group 1		Group 2	
		Y = 1 vs. Y = 2–3		Y = 1–2 vs. Y = 3	
		*β* (*P*-value)	OR (95% CI of OR)	*β* (*P*-value)	OR (95% CI of OR)
Age	–	−0.016 (0.077)^*^	0.98 (0.97–1.00)	−0.016(0.077)^*^	0.98 (0.97–1.00)
Impact of fertility attitudes	-	0.029 (0.264)	1.03 (0.98–1.08)	−0.039 (0.095)^*^	0.96 (0.92–1.01)
Impact of childcare challenges	-	0.078 (0.006)^***^	1.08 (1.02–1.14)	0.078 (0.006)^***^	1.08 (1.02–1.14)
Impact of social and family expectations	-	−0.087 (0.007)^***^	0.92 (0.86–0.98)	−0.018 (0.540)	0.98 (0.93–1.04)
Educational level (reference: High school/Vocational High school/Technical secondary school)	Junior high school or below	0.277 (0.149)	1.32 (0.91–1.92)	0.277 (0.149)	1.32 (0.91–1.92)
	College / Bachelor’s degree	−0.086 (0.563)	0.92 (0.69–1.23)	−0.086 (0.563)	0.92 (0.69–1.23)
	Postgraduate and above	−0.232 (0.264)	0.79 (0.53–1.19)	−0.232 (0.264)	0.79 (0.53–1.19)
Marital status (reference: unmarried)	Married with no children	−0.417 (0.054)^*^	0.66 (0.43–1.01)	−0.417 (0.054)^*^	0.66 (0.43–1.01)
	Married with children	0.524 (0.007)^***^	1.69 (1.16–2.46)	0.013 (0.946)	1.01 (0.71–1.45)
Occupation (reference: Civil servant, public institution or state-owned enterprise personnel)	Foreign/private enterprise personnel/Self-employed	−0.466 (0.009)^***^	0.63 (0.44–0.89)	0.120 (0.481)	1.13 (0.81–1.57)
	Migrant worker/Peasant	−0.173 (0.350)	0.84 (0.59–1.21)	−0.173 (0.350)	0.84 (0.59–1.21)
	Student	−0.084 (0.687)	0.92 (0.61–1.39)	−0.084 (0.687)	0.92 (0.61–1.39)
	Freelance work	−0.013 (0.949)	0.99 (0.65–1.49)	−0.013 (0.949)	0.99 (0.65–1.49)
	Other practitioners/Unemployed	0.386 (0.006)^***^	1.47 (1.12–1.94)	0.386 (0.006)^***^	1.47 (1.12–1.94)
Self-Rated health (reference: very healthy)	Suboptimal health	0.296 (0.008)^***^	1.35 (1.08–1.67)	−0.063 (0.522)	0.94 (0.77–1.14)
Intended number of children (reference: 1)	0	−0.053 (0.771)	0.95 (0.66–1.36)	−0.053 (0.771)	0.95 (0.66–1.36)
	2	−0.016(0.899)	0.98 (0.77–1.26)	−0.339 (0.003)^***^	0.71 (0.57–0.89)
	≥3	−0.616 (0.010)^***^	0.54 (0.34–0.86)	−0.616 (0.010)^***^	0.54 (0.34–0.86)
Career-fertility conflict (reference: balancing both)	Career prioritized	0.021 (0.829)	1.02 (0.84–1.24)	0.021 (0.829)	1.02 (0.84–1.24)
	Childbearing prioritized	−0.124 (0.461)	0.88 (0.64–1.23)	−0.124 (0.461)	0.88 (0.64–1.23)
Only-child status (reference: no)	Yes	−0.283 (0.026)^**^	0.75 (0.59–0.97)	−0.019 (0.874)	0.98 (0.78–1.24)
Household size (reference: 4)	1–2	−0.061 (0.766)	0.94 (0.63–1.41)	−0.061 (0.766)	0.94 (0.63–1.41)
	3	−0.439(<0.001)^***^	0.64 (0.52–0.80)	−0.439(<0.001)^***^	0.64 (0.52–0.80)
	5	−0.108 (0.406)	0.90 (0.69–1.16)	−0.108 (0.406)	0.90 (0.69–1.16)
	≥6	−0.016(0.910)	0.98 (0.74–1.30)	−0.016(0.910)	0.98 (0.74–1.30)
Residence (reference: Urban)	Rural	0.408 (0.001)^***^	1.50 (1.17–1.93)	0.408 (0.001)^***^	1.50 (1.17–1.93)
Monthly household income (reference: 6,001–8,000 RMB)	≤4,000 RMB	−0.134 (0.389)	0.87 (0.65–1.19)	−0.134 (0.389)	0.87 (0.65–1.19)
	4,001–6,000 RMB	−0.282 (0.049)^**^	0.75 (0.57–1.00)	−0.282 (0.049)^**^	0.75 (0.57–1.00)
	8,001–10,000 RMB	−0.210 (0.150)	0.81 (0.61–1.08)	−0.210 (0.150)	0.81 (0.61–1.08)
	10,001–15,000 RMB	−0.062 (0.671)	0.94 (0.70–1.25)	−0.062 (0.671)	0.94 (0.70–1.25)
	>15,000 RMB	−0.078 (0.607)	0.92 (0.69–1.25)	−0.078 (0.607)	0.92 (0.69–1.25)
Awareness of fertility policy (reference: Clear)	Incorrect	−0.087 (0.336)	0.92 (0.77–1.09)	−0.087 (0.336)	0.92 (0.77–1.09)
	Unclear	−0.315 (0.143)	0.73 (0.48–1.11)	0.535 (0.004)^***^	1.71 (1.18–2.47)
Intercept	–	1.869(<0.001)^***^	6.48 (3.08–13.65)	0.249 (0.503)	1.28 (0.62–2.66)
Sample size	–	2090		2090	

1. β and *P*-value are rounded to three decimals; OR and 95% CI are rounded to two decimals. 2. Significance levels: *p* < 0.10 (*), *p* < 0.05 (**), *p* < 0.01 (***). 3. The independent variables represent category levels of each factor, while the dependent variable Y denotes ideal birth spacing: Y = 1 (0–2 years), Y = 2 (3–4 years) and Y = 3 (≥5 years or no specific preference); comparisons: Y = 1 vs. Y = 2–3 and Y = 1–2 vs. Y = 3.

**Table 3:** Average marginal effects from the generalized ordered logit model for ideal birth spacing.

Variables	Category	Short ideal spacing		Medium ideal spacing		Long ideal spacing	
		Estimated value (SE)	*P*-value	Estimated value (SE)	*P*-value	Estimated value (SE)	*P*-value
Age	–	0.003 (0.001)	0.077^*^	0.001 (0.000)	0.084^*^	−0.003 (0.002)	0.077^*^
Impact of fertility attitudes	–	−0.005 (0.004)	0.264	0.013 (0.005)	0.011^**^	−0.008 (0.005)	0.094^*^
Impact of childcare challenges	–	−0.012 (0.005)	0.006^***^	−0.004 (0.002)	0.009^***^	0.016(0.006)	0.006^***^
Impact of social and family expectations	–	0.014 (0.005)	0.007^***^	−0.010 (0.006)	0.091^*^	−0.004 (0.006)	0.540
Educational level (reference: High school/Vocational High school/Technical secondary school)	Junior high school or below	−0.039 (0.026)	0.139	−0.022 (0.017)	0.197	0.061 (0.043)	0.154
	College / Bachelor’s degree	0.013 (0.023)	0.557	0.005 (0.009)	0.592	−0.018 (0.032)	0.566
	Postgraduate and above	0.038 (0.034)	0.266	0.010 (0.009)	0.280	−0.048 (0.043)	0.262
Marital status (reference: Unmarried)	Married with no children	0.085 (0.044)	0.054^*^	−0.003 (0.007)	0.708	−0.082 (0.042)	0.052^*^
	Married with children	−0.087 (0.034)	0.012^**^	0.084 (0.024)	0.001^***^	0.003 (0.039)	0.946
Occupation (reference: Civil servant, public institution or state-owned enterprise personnel)	Foreign/private enterprise personnel/Self-employed	0.082 (0.033)	0.014^**^	−0.107 (0.034)	0.002^***^	0.025 (0.036)	0.486
	Migrant worker/Peasant	0.028 (0.031)	0.362	0.007 (0.006)	0.246	−0.035 (0.036)	0.340
	Student	0.013 (0.034)	0.629	0.004 (0.008)	0.652	−0.017 (0.042)	0.684
	Freelance work	0.002 (0.033)	0.949	0.001 (0.010)	0.948	−0.003 (0.043)	0.949
	Other practitioners/Unemployed	−0.054 (0.019)	0.004^***^	−0.031 (0.013)	0.021^**^	0.085 (0.031)	0.007^***^
Self-Rated health (reference: Very healthy)	Suboptimal health	−0.047 (0.018)	0.009^***^	0.061 (0.021)	0.005^***^	−0.013 (0.021)	0.523
Intended number of children (reference: 1)	0	0.008 (0.029)	0.773	0.003 (0.011)	0.760	−0.012 (0.040)	0.770
	2	0.002 (0.019)	0.899	0.069 (0.023)	0.002^***^	−0.071 (0.024)	0.002^***^
	≥3	0.111 (0.048)	0.020^**^	0.012 (0.008)	0.105	−0.123 (0.043)	0.004^***^
Career-fertility conflict (reference: Balancing both)	Career prioritized	−0.003 (0.015)	0.829	−0.001 (0.005)	0.831	0.004 (0.021)	0.830
	Childbearing prioritized	0.020 (0.028)	0.472	0.005 (0.006)	0.377	−0.025 (0.034)	0.453
Only-child status (reference: No)	Yes	0.047 (0.022)	0.032^**^	−0.043 (0.024)	0.082^*^	−0.004 (0.025)	0.874
Household size (reference: 4)	1–2	0.009 (0.030)	0.769	0.004 (0.014)	0.756	−0.013 (0.045)	0.765
	3	0.071 (0.018)	<0.001^***^	0.019 (0.006)	0.001^***^	−0.091 (0.023)	<0.001^***^
	5	0.016(0.020)	0.411	0.008 (0.009)	0.392	−0.024 (0.028)	0.404
	≥6	0.002 (0.021)	0.910	0.001 (0.011)	0.910	−0.004 (0.032)	0.910
Residence (reference: Urban)	Rural	−0.061 (0.018)	0.001^***^	−0.028 (0.011)	0.010^***^	0.089 (0.028)	0.002^***^
Monthly household income (reference: 6,001–8,000 RMB)	≤4,000 RMB	0.020 (0.024)	0.392	0.008 (0.009)	0.383	−0.029 (0.033)	0.387
	4,001–6,000 RMB	0.045 (0.023)	0.049^**^	0.014 (0.008)	0.071^*^	−0.059 (0.030)	0.048^**^
	8,001–10,000 RMB	0.033 (0.023)	0.151	0.012 (0.008)	0.165	−0.044 (0.031)	0.150
	10,001–15,000 RMB	0.009 (0.022)	0.671	0.004 (0.010)	0.672	−0.013 (0.032)	0.671
	>15,000 RMB	0.012 (0.023)	0.608	0.005 (0.010)	0.607	−0.017 (0.033)	0.607
Awareness of fertility policy (reference: Clear)	Incorrect	0.014 (0.014)	0.335	0.005 (0.005)	0.346	−0.018 (0.019)	0.337
	Unclear	0.052 (0.038)	0.167	−0.173 (0.037)	<0.001^***^	0.121 (0.044)	0.006^***^

1. The numbers in the table [e.g., 0.003 (0.001)] represent the estimated marginal effects and their standard errors. 2. Significance levels: *p* < 0.10 (*), *p* < 0.05 (**), *p* < 0.01 (***). 3. Short ideal spacing: 0–2 years, Medium ideal spacing: 3–4 years, Long ideal spacing: ≥5 years or no specific preference.

##### 3.2.3.1 Factors with uniform effects across spacing thresholds

Variables in this section satisfied the proportional odds assumption, indicating uniform effects across thresholds.

###### 3.2.3.1.1 Cumulative odds ratios

Among demographic and socioeconomic determinants, occupation, residence and household income showed stable effects. Women in other occupations or who were unemployed were associated with higher odds of preferring long spacing (OR = 1.47, 95% CI: 1.12–1.94; *p* = 0.006). Rural residents were also associated with higher odds of preferring long spacing compared with urban residents (OR = 1.50, 95% CI: 1.17–1.93; *p* = 0.001). Compared with households earning 6,001–8,000 RMB per month, those earning 4,000–6,000 RMB were associated with lower odds of preferring long spacing (OR = 0.75, 95% CI: 0.57–1.00; *p* = 0.049).

Family structure, fertility intentions and perceived childcare challenges were associated with spacing preferences. A higher perceived impact of childcare challenges was associated with higher odds of preferring long spacing (OR = 1.08, 95% CI: 1.02–1.14; *p* = 0.006). Childless married women were suggestively associated with lower odds of preferring short spacing compared with unmarried women (OR = 0.66, 95% CI: 0.43–1.01; *p* = 0.054). Intending to have three or more children were associated with lower odds of preferring long spacing than those intending only one child (OR = 0.54, 95% CI: 0.34–0.86; *p* = 0.010). Women from three-member households were associated with lower odds of preferring long spacing than those from four-member households (OR = 0.64, 95% CI: 0.52–0.80; *p* < 0.001).

###### 3.2.3.1.2 Average marginal effects

The AMEs showed that women in other occupations or who were unemployed showed an 8.5 pp higher probability of long spacing (AME = 0.085, *p* = 0.007). Rural residents had an 8.9 pp higher probability of long spacing (AME = 0.089, *p* < 0.001). By contrast, the 4,000–6,000 RMB income group showed a 5.9 pp lower probability of long spacing (AME = −0.059, *p* = 0.048). Each unit increase in perceived childcare challenges showed a 1.6 pp higher probability of long spacing (AME = 0.016, *p* = 0.006). Childless married women showed an 8.5 pp higher probability of short spacing (AME = 0.085, *p* = 0.054). Intending to have three or more children showed an 11.1 pp higher probability of short spacing (AME = 0.111, *p* = 0.020). Those from three-member households showed a 9.1 pp lower probability of long spacing (AME = −0.091, *p* < 0.001).

##### 3.2.3.2 Factors primarily influencing short ideal birth spacing

Variables in this section did not satisfy the proportional odds assumption. Threshold specific effects were estimated using the gologit model.

###### 3.2.3.2.1 Cumulative odds ratios

Social factors and employment characteristics shaped preferences for short spacing. A higher perceived impact of social and family expectations was associated with higher odds of preferring short spacing (OR = 0.92, 95% CI: 0.86–0.98; *p* = 0.007). Women employed in foreign, private or self-employed sectors were associated with higher odds of preferring short spacing compared with those in government or public institutions (OR = 0.63, 95% CI: 0.44–0.89; *p* = 0.009).

Individual and family background factors also influenced preferences. Married women with children were associated with lower odds of preferring short spacing compared with unmarried women (OR = 1.69, 95% CI: 1.16–2.46; *p* = 0.007). Compared with women in very good health, those reporting suboptimal health were associated with lower odds of preferring short spacing (OR = 1.35, 95% CI: 1.08–1.67; *p* = 0.008). Only-child women were associated with higher odds of preferring short spacing than non-only-child women (OR = 0.75, 95% CI: 0.59–0.97; *p* = 0.026).

###### 3.2.3.2.2 Average marginal effects

Each unit increase in perceived social and family expectations showed a 1.4 pp higher probability of short spacing (AME = 0.014, *p* = 0.007). Women employed in foreign/private enterprises or self-employed sectors showed an 8.2 pp higher probability of short spacing (AME = 0.082, *p* = 0.014). Married women with children showed an 8.7 pp lower probability of short spacing (AME = −0.087, *p* = 0.012). Women with suboptimal health showed a 4.7 pp lower probability of short spacing (AME = −0.047, *p* = 0.008). Only-child women showed a 4.7 pp higher probability of short spacing (AME = 0.047, *p* = 0.032).

##### 3.2.3.3 Factors mainly influencing long ideal birth spacing

Variables in this section also did not satisfy the proportional odds assumption. Threshold specific effects were estimated using the gologit model.

###### 3.2.3.3.1 Cumulative odds ratios

Fertility intentions and policy awareness influenced preferences for longer spacing. Intending to have two children was associated with lower odds of preferring long spacing compared with intending only one child (OR = 0.71, 95% CI: 0.57–0.89; *p* = 0.003). Compared with women who had clear knowledge of fertility policies, those who were unclear about them were associated with higher odds of preferring long spacing (OR = 1.71, 95% CI: 1.18–2.47; *p* = 0.004).

###### 3.2.3.3.2 Average marginal effects

Intending to have two children showed a 7.1 pp lower probability of long spacing (AME = −0.071, *p* = 0.002). By contrast, those who were unclear about fertility policies showed a 12.1 pp higher probability of long spacing (AME = 0.121, *p* = 0.006).

## 4 Discussion

This study examined the ideal birth spacing preferences and their associated factors among reproductive-aged women in Shandong Province. The findings revealed a general preference for medium spacing (3–4 years) over short or long spacing. The perceived impact of social and family expectations and the perceived impact of childcare challenges were associated with ideal birth spacing, although the effect sizes were relatively small. By contrast, fertility policy awareness, intended number of children, marital status, residence, and occupational type showed larger effect sizes.

### 4.1 Discussion of ideal birth spacing preferences

The overall preference for medium spacing (3–4 years) observed in this study is consistent with findings from other regions. International evidence indicates that women generally prefer medium or longer birth spacing, whereas very short spacing (less than 24 months) is less common (29, 30). The median ideal birth spacing typically exceeds 30 months (29, 30). This pattern may be influenced by cultural norms, socioeconomic conditions, and policy environments, which shape women’s perceptions of childbearing demands and timing (30–33). Women may balance maternal recovery, childcare responsibilities, and a limited reproductive window when considering their ideal birth spacing. Policy environment may further affect these preferences by shaping the perceived costs and support associated with childbearing. Overall, medium spacing appears to reflect a balanced consideration of multiple factors in reproductive decision-making.

### 4.2 Discussion of factors influencing ideal birth spacing

#### 4.2.1 Influence of social and family expectations, and childcare challenges on ideal birth spacing

The results indicated that women’s ideal birth spacing was associated with both social and family expectations and childcare challenges. These findings are consistent with evidence from multiple countries showing that social normative pressures and childcare burdens jointly shape fertility intentions and birth timing (34–38). The influences likely operate through distinct pathways. On the one hand, perceived social and family expectations, transmitted through spouses, kin, friends, and broader social networks, shape perceptions of appropriate childbearing timing (39). In this context, women are more likely to view childbearing as a task that should be completed earlier, leading to a preference for shorter ideal birth spacing (40). On the other hand, when women perceive greater childcare challenges, they may adjust their fertility plans to allocate more time, effort, and resources to childrearing. This adjustment may also be shaped by career development considerations, as women may delay childbearing to accommodate critical periods in their professional advancement (41). Such adjustments are associated with longer ideal birth spacing.

The magnitude of these associations may vary depending on the availability of public childcare support. When formal support is sufficient, reliance on external resources may weaken the influence of social and family expectations. When support is limited, family-based resources become more important, and fertility decisions are more strongly shaped by partners, parents, and broader social networks (42–45). Thus, women’s ideal birth spacing reflects a negotiated balance between the push of social expectations and the pull of childcare challenges, a dynamic consideration in fertility planning.

#### 4.2.2 Influence of marital status on ideal birth spacing

Marital status serves as a key marker of life stages associated with distinct fertility spacing preferences. Specifically, married women without children tended to prefer shorter spacing, whereas the opposite pattern was observed among married women with children. The preference for shorter spacing among married women without children may reflect that marriage itself initiates fertility planning. First, marriage provides institutional support for childbearing, enabling women to access statutory benefits such as maternity leave and spousal childcare allowances, while unmarried mothers may face more limited access to such benefits. Second, partners can share the time and care responsibilities associated with prenatal and postnatal childcare, thereby reducing the individual burden of childbearing. This shared arrangement may also encourage relatively earlier childbearing to reduce risks arising from advanced maternal age and career uncertainty (46–48). However, this pattern appears to shift when women become mothers. The concrete demands of childcare, such as daily care, education, and household financial pressure, are likely to lead them to reassess their reproductive timing and favor longer or more moderate spacing (36). From a life course perspective, these stage-specific shifts illustrate how fertility preferences evolve across marriage and childbearing transitions, highlighting the dynamic process of ideal birth spacing formation (49, 50). Overall, these findings suggest that tailored support at different life stages may help women better manage their fertility timing.

#### 4.2.3 Influence of intended number of children on ideal birth spacing

Compared with women planning only one child, those planning two children tend to prefer a moderate spacing, whereas those planning three or more children tend to favor shorter spacing. This pattern may be partly explained by the postponement of first marriage and first birth, which substantially compresses the reproductive window available for childbearing (51). Consequently, women planning three or more children often face acute time constraints over the course of their reproductive lives (5, 52). Under such conditions, fertility planning is limited to a narrower range of feasible spacing options, making shorter birth spacing more likely among women planning larger families. This tendency may also reflect considerations related to maternal and neonatal health. Evidence indicates that short birth spacing may increase the risks of adverse outcomes, including insufficient maternal recovery, preterm birth, and low birth weight (53). For women with greater temporal flexibility, extending spacing may serve as a feasible strategy to mitigate such risks. For those intending to have three or more children, this strategy is often constrained in practice. Thus, this pattern may be better understood as a pragmatic compromise in which objective time constraints outweigh individual preferences or intentions.

By contrast, women planning two children generally face less time pressure. Their preference for moderate spacing reflects a more balanced consideration of multiple factors, including maternal recovery, the transition of childcare responsibilities between two children, and potential career progression (53, 54). Together, these findings indicate that the intended number of children may shape women’s ideal birth spacing by structuring distinct temporal horizons in fertility planning.

#### 4.2.4 Influence of fertility policy awareness on ideal birth spacing

Fertility policy awareness is associated with women’s ideal birth spacing decisions. Women with a clear understanding of fertility policy can incorporate policy supports (such as maternity allowances, parental leave and childcare services) into their personal plans. This understanding may reduce perceived uncertainty regarding the costs and risks of childbearing, making women more likely to plan shorter ideal birth spacing and utilize available policy support (55). Conversely, women with unclear awareness often perceive higher costs and risks associated with childbearing (56). They may tend to delay fertility decisions and prefer longer ideal birth spacing to reduce potential risks. Similar patterns have been observed in Japan, where limited fertility knowledge is linked to delayed childbearing, while better understanding is associated with more favorable fertility intentions (57, 58).

Two considerations are important for interpreting this association. First, policy awareness is not equivalent to institutional trust. When confidence in policy implementation is limited, its role in reducing uncertainty may be constrained (59). Second, policy awareness is closely related to women’s socioeconomic status and access to information. Women with higher socioeconomic status and better access to information are more likely to be informed and better able to translate policy support into fertility planning (60, 61). Thus, the observed association between policy awareness and ideal birth spacing may partly reflect underlying socioeconomic and informational differences rather than an independent effect of awareness alone. Overall, fertility policy awareness appears to play a role in shaping women’s ideal birth spacing.

### 4.3 Strengths, limitations and implications

This study has several notable strengths. First, the large sample of 2,090 valid questionnaires provided adequate statistical power for the analyses. Second, the multistage sampling design ensured that the participants were representative of women of reproductive age in Shandong Province. Third, the questionnaire demonstrated high reliability and validity, ensuring data quality. Fourth, by examining women’s perceptions of ideal birth spacing, the study offers a cognitive perspective on fertility and provides insights into how reproductive decisions are formed.

This study also has several limitations. Firstly, the cross-sectional design allows identification of associations but cannot establish causal relationships; future studies are needed to use longitudinal designs to explore causality. Secondly, the study included only participants from Shandong Province, so the results may not apply to other regions. Rural residents were underrepresented and women working in government or public institutions were overrepresented, which could limit generalizability. Future studies could include participants from multiple provinces and aim for a more balanced sample to improve representativeness. Thirdly, although multiple factors were selected based on the social ecological model, some important influences may have been missed. Future research could consider detailed policy knowledge or access to community childcare resources. Fourthly, the categorization of ideal birth spacing is relatively coarse and may not fully capture heterogeneity within response categories. In particular, the grouping of “≥5 years” and “no specific preference” may have led to some loss of information and reduced granularity in the measurement of the ordinal outcome. Future studies could consider more refined category definitions or alternative modeling strategies. Fifthly, although a multi-stage sampling design was employed, potential clustering effects, design effects, and sampling weights were not explicitly accounted for in the statistical analyses. This may affect the precision of the estimates. Future studies could benefit from incorporating sampling weights and accounting for design effects in the statistical analyses. Additionally, using a *p* < 0.10 threshold for variable selection may have increased the risk of including weak associations. Future studies with larger samples are recommended to further validate these findings. Finally, women who intend to have only one child may have distinct considerations regarding birth spacing, which could introduce heterogeneity into the observed preferences. Future studies could conduct separate or comparative analyses of women intending to have only one child, to further examine the determinants of their birth spacing preferences and to assess whether their decision-making processes differ systematically from those of women intending to have two or more children.

Several policy implications may be tentatively drawn from this study. First, strengthening childcare services may help reduce family burdens and support women’s fertility planning. Second, flexible and phased support measures may be worth considering to accommodate differences in intended number of children. Third, improving information infrastructure, education, and employment opportunities in rural areas might enhance women’s reproductive autonomy. Fourth, fertility policy information could be disseminated through multiple channels to improve clarity and accessibility. Given the cross-sectional nature of this study, these implications should be interpreted with caution and treated as suggestive rather than prescriptive.

## 5 Conclusion

This study found that reproductive-aged women in Shandong Province have diverse preferences regarding ideal birth spacing, with a substantial proportion preferring a medium spacing of 3–4 years. These preferences are shaped by a combination of individual, family and sociocultural factors. The findings suggest that fertility support policies should focus on reducing childcare burdens, improve policy awareness, and provide stage-specific support. Such targeted approaches may help women make more informed and effective fertility decisions.

## Data Availability

The raw data supporting the conclusions of this article will be made available by the authors, without undue reservation.
