# Supplementary material for: Ideal birth spacing between the first and second child among reproductive-aged women in Shandong Province, China
Source: Front Public Health. 2026 Jul 13;14:1842402. doi: 10.3389/fpubh.2026.1842402 (PMC13426076; doi:10.3389/fpubh.2026.1842402)
Supplement: Supplementary file 1 [file Table_1.DOCX]

| **Supplementary Table 1 Details of Questionnaire Items and Dimensional Indicators** | | |
| --- | --- | --- |
| Dimensional indicators | Options | Item |
| Impact of fertility attitudes | Q35-1 | Does the traditional fertility view of “raising sons for old-age support” influence your decision to have two children? |
|  | Q35-2 | Does the traditional fertility view of “continuing the family line” influence your decision to have two children? |
|  | Q35-3 | Does the traditional fertility view of “the more children, the greater the blessing” influence your decision to have two children? |
|  | Q35-4 | Do emerging parenting concepts such as “pyramid-style parenting” and “elite-style parenting” influence your decision not to have two children? |
|  | Q35-5 | Does the fertility view focusing on emotional comfort through fulfilling family affection needs influence your decision to have two children? |
| Impact of childcare challenges | Q37 | Does the issue of childcare before preschool affect your decision to have two children? |
|  | Q39 | Does the preschool drop-off and pick-up arrangement affect your decision to have two children? |
|  | Q41 | Does the primary school drop-off and pick-up arrangement affect your decision to have two children? |
|  | Q43 | Do school-related assignments and supervision requirements (such as check-ins) during children’s school years affect your decision to have two children? |
| Impact of social and familial expectations | Q33-1 | Does your husband’s expectation of having a second child affect your decision to have two children? |
|  | Q33-2 | Do your parents-in-law’s expectations of having a second child affect your decision to have two children? |
|  | Q33-3 | Do your parents’ expectations of having a second child affect your decision to have two children? |

| **Supplementary Table 1 (continued)** |  |  |
| --- | --- | --- |
| Dimensional indicators | Options | Item |
|  | Q33-4 | Do your colleagues’, relatives’, neighbors’ and friends’ attitudes toward having a second child affect your decision to have two children? |
|  | Q33-5 | Does society’s expectation of having a second child affect your decision to have two children? |

| **Supplementary Table 2 Univariate Analysis of Factors Influencing Ideal Birth Spacing** | | | | | | | |
| --- | --- | --- | --- | --- | --- | --- | --- |
| Characteristic | Category | Frequency（%） | Ideal Birth Spacing [n (%) / Median (IQR)] | | | χ^2^/H | *P*-value |
|  |  |  | 0–2 years | 3–4 years | ≥5 years or no specific preference |  |  |
| Participates | - | 2090 (100.00) | 441 (21.10) | 968 (46.32) | 681 (32.58) | - | - |
| Age | - | 2090 (100.00) | 34 (27, 40) | 35 (30, 40) | 34 (28, 39) | 23.36 | <0.001^*^ |
| Impact of fertility attitudes | - | 2090 (100.00) | 0.09 (−3.04, 2.09) | 0.65 (−2.27, 2.27) | −0.65 (−3.04, 2.01) | 9.59 | 0.008^*^ |
| Impact of childcare challenges | - | 2090 (100.00) | 0.15 (−1.53, 1.27) | 0.21 (−1.17, 1.26) | 0.27 (−1.20, 1.36) | 4.04 | 0.133^*^ |
| Impact of social and family expectations | - | 2090 (100.00) | 0.17 (−1.80, 1.241) | −0.12 (−1.80, 1.24) | −0.43 (−2.10, 0.94) | 4.24 | 0.120^*^ |
| Educational level | Junior high school or below | 191 (9.14) | 33 (17.28) | 77 (40.31) | 81 (42.41) | 17.71 | 0.007^#^ |
|  | High school/Vocational High school/Technical secondary school | 274 (13.11) | 60 (21.90) | 118 (43.07) | 96 (35.04) |  |  |
|  | College/Bachelor's degree | 1416 (67.75) | 305 (21.54) | 658 (46.47) | 453 (31.99) |  |  |
|  | Postgraduate and above | 209 (10.00) | 43 (20.57) | 115 (55.02) | 51 (24.40) |  |  |
| Marital status | Unmarried | 370 (17.70) | 94 (25.41) | 128 (34.59) | 148 (40.00) | 40.53 | <0.001^#^ |
|  | Married with no children | 185 (8.85) | 58 (31.35) | 80 (43.24) | 47 (25.41) |  |  |
|  | Married with children | 1535 (73.44) | 289 (18.83) | 760 (49.51) | 486 (31.66) |  |  |
| Awareness of fertility policy | Incorrect | 1066 (51.01) | 224 (21.01) | 523 (49.06) | 319 (29.92) | 29.05 | <0.001^#^ |
|  | Clear | 886 (42.39) | 183 (20.65) | 409 (46.16) | 294 (33.18) |  |  |
|  | Unclear | 138 (6.60) | 34 (24.64) | 36 (26.09) | 68 (49.28) |  |  |

| **Supplementary Table 2 (continued)** | | | | | | | |
| --- | --- | --- | --- | --- | --- | --- | --- |
| Characteristic | Category | Frequency（%） | Ideal Birth Spacing [n (%) / Median (IQR)] | | | χ^2^/H | *P*-value |
|  |  |  | 0–2 years | 3–4 years | ≥5 years or no specific preference |  |  |
| Occupation | Civil servant, public institution or state-owned enterprise personnel | 1054 (50.43) | 217 (20.59) | 541 (51.33) | 296 (28.08) | 42.59 | <0.001^#^ |
|  | Foreign/private enterprise personnel/Self-employed | 221 (10.57) | 63 (28.51) | 83 (37.56) | 75 (33.94) |  |  |
|  | Migrant worker/Peasant | 228 (10.91) | 46 (20.18) | 103 (45.18) | 79 (34.65) |  |  |
|  | Student | 191 (9.14) | 47 (24.61) | 67 (35.08) | 77 (40.31) |  |  |
|  | Freelance work | 111 (5.31) | 21 (18.92) | 53 (47.75) | 37 (33.33) |  |  |
|  | Other practitioners/Unemployed | 285 (13.64) | 47 (16.49) | 121 (42.46) | 117 (41.05) |  |  |
| Self-Rated health | Very healthy | 867 (41.48) | 216 (24.91) | 366 (42.21) | 285 (32.87) | 15.63 | <0.001^#^ |
|  | Suboptimal health | 1223 (58.52) | 225 (18.40) | 602 (49.22) | 396 (32.38) |  |  |
| Intended number of children | 0 | 138 (6.60) | 33 (23.91) | 50 (36.23) | 55 (39.86) | 25.70 | <0.001^#^ |
|  | 1 | 977 (46.79) | 207 (21.19) | 424 (43.40) | 346 (35.41) |  |  |
|  | 2 | 901 (43.11) | 177 (19.64) | 464 (51.50) | 260 (28.86) |  |  |
|  | ≥3 | 74 (3.54) | 24 (32.43) | 30 (40.54) | 20 (27.03) |  |  |
| Career-fertility conflict | Career prioritized | 666 (31.86) | 135 (20.27) | 295 (44.29) | 236 (35.44) | 4.49 | 0.344^#^ |
|  | Childbearing prioritized | 152 (7.27) | 36 (23.68) | 73 (48.03) | 43 (28.29) |  |  |
|  | Balancing both | 1272 (60.87) | 270 (21.23) | 600 (47.17) | 402 (31.60) |  |  |

| **Supplementary Table 2 (continued)** | | | | | | | |
| --- | --- | --- | --- | --- | --- | --- | --- |
| Characteristic | Category | Frequency（%） | Ideal Birth Spacing [n (%) / Median (IQR)] | | | χ^2^/H | *P*-value |
|  |  |  | 0–2 years | 3–4 years | ≥5 years or no specific preference |  |  |
| Only-child status | Yes | 485 (23.20) | 137 (28.25) | 201 (41.44) | 147 (30.31) | 19.53 | <0.001^#^ |
|  | No | 1605 (76.80) | 304 (18.94) | 767 (47.79) | 534 (33.27) |  |  |
| Household size | 1-2 | 140 (6.70) | 41 (29.29) | 53 (37.86) | 46 (32.86) | 31.81 | <0.001^#^ |
|  | 3 | 686 (32.82) | 180 (26.24) | 306 (44.61) | 200 (29.15) |  |  |
|  | 4 | 672 (32.13) | 124 (18.45) | 308 (45.83) | 240 (35.71) |  |  |
|  | 5 | 331 (15.83) | 55 (16.62) | 168 (50.76) | 108 (32.63) |  |  |
|  | ≥6 | 261 (12.49) | 41 (15.71) | 133 (50.96) | 87 (33.33) |  |  |
| Residence | Urban areas | 1563 (74.83) | 350 (22.39) | 746 (47.73) | 467 (29.88) | 21.50 | <0.001^#^ |
|  | Rural areas | 527 (25.17) | 91 (17.27) | 222 (42.13) | 214 (40.61) |  |  |
| Monthly household income | ≤4,000 RMB | 308 (14.73) | 65 (21.10) | 132 (42.86) | 111 (36.04) | 8.37 | 0.593^#^ |
|  | 4,001-6,000 RMB | 374 (17.89) | 84 (22.46) | 168 (44.92) | 122 (32.62) |  |  |
|  | 6,001–8,000 RMB | 356 (17.03) | 63 (17.70) | 165 (46.35) | 128 (35.96) |  |  |
|  | 8,001–10,000 RMB | 342 (16.36) | 74 (21.64) | 166 (48.54) | 102 (29.82) |  |  |
|  | 10,001–15,000 RMB | 363 (17.37) | 76 (20.94) | 173 (47.66) | 114 (31.40) |  |  |
|  | >15,000 RMB | 347 (16.60) | 79 (22.77) | 164 (47.26) | 104 (29.97) |  |  |
| Notes:  ^*^：Kruskal–Wallis H;  ^#^：Chi-square test | | | | | | | |

| **Supplementary Table 3 Collinearity test of independent variables** | | |
| --- | --- | --- |
| Characteristic | VIF | Tolerance |
| Age | 2.12 | 0.4706 |
| Impact of fertility attitudes | 1.40 | 0.7151 |
| Impact of childcare challenges | 1.31 | 0.7659 |
| Impact of social and familial expectations | 1.51 | 0.6641 |
| Educational level | 1.68 | 0.5966 |
| Marital status | 2.10 | 0.4759 |
| Occupation | 1.26 | 0.7957 |
| Self-Rated health | 1.04 | 0.9651 |
| Intended number of children | 1.36 | 0.7349 |
| Career–fertility conflict | 1.13 | 0.8880 |
| Only-child status | 1.10 | 0.9129 |
| Household size | 1.20 | 0.8311 |
| Residence | 1.52 | 0.6557 |
| Monthly household income | 1.35 | 0.7410 |
| Awareness of fertility policy | 1.06 | 0.9474 |
| Note：VIF：variance inflation factor |  |  |

| **Supplementary Table 4 Model Goodness of Fit Test** | | | |
| --- | --- | --- | --- |
| Model | Pseudo R^2^ | AIC | BIC |
| Ologit | 0.025 | 4348.72 | 4546.29 |
| Gologit | 0.045 | 4280.20 | 4522.93 |
